# ‘He would by no means risque his Reputation’: patient and doctor shame in Daniel Turner's *De Morbis Cutaneis* (1714) and *Syphilis* (1717)

**DOI:** 10.1136/medhum-2016-011057

**Published:** 2017-01-17

**Authors:** Emily Cock

## Abstract

This article offers a historical corollary to the examination of shame in medical practice by considering the negotiation of shame in the treatment of a stigmatised disease at a time in which surgeons themselves occupied a highly ambivalent social position. It will focus on case studies provided by Daniel Turner (1667–1741), prominent surgeon and later member of the College of Physicians, in his textbooks *De Morbis Cutaneis. A Treatise of Diseases Incident to the Skin* (1714) and *Syphilis. A Practical Dissertation on the Venereal Disease* (1717). Turner demonstrates an awareness of the precarious position of both the surgeon and the syphilitic, and devotes significant portions of his text to advising the trainee surgeon on how to manage patients' reticence over disclosure of symptoms, expectations for cure and impudence towards medical authority. In turn, the trainee must manage his own reputation as a moral and medical authority who can treat all distempers, yet without condoning or facilitating the shameful behaviours associated with a sexual disease. Furthermore, shaming plays a key role in enabling Turner to fashion an ideal patient whose successful cure will both respond to and build the surgeon's medical authority and that of the medical field in general.

This article offers a historical perspective to the consideration of shame and medicine by examining the negotiation of shame by a prominent early eighteenth-century surgeon and physician, Daniel Turner (1667–1741). This was a period in which surgeons themselves occupied a highly ambivalent social position and their customers enjoyed greater choice of domestic and market medical care. Physicians and surgeons therefore had to employ a variety of methods to attract and keep customers, and to ensure their compliance. I draw on the case studies and further notes provided in two of Turner's mid-career texts: the first, *De Morbis Cutaneis. A Treatise of Diseases Incident to the Skin* (1714), was the first book solely dedicated to diseases of the skin;^1^ the second, *Syphilis. A Practical Dissertation on the Venereal Disease* (1717),^2^ represented a significant contribution to a much more well-populated field. Each text was reprinted several times in the first half of the eighteenth century.^i^ In these texts, shaming plays a key role in enabling Turner to fashion an ideal patient whose successful cure will both respond to and build the practitioner's medical authority, and that of professional medicine in general. Historians of emotions hold the eighteenth century as a transitional period for how Western culture experienced, understood and used shame,^4–7^ and in his interactions with patients Turner appears to accord with the turn to shame as an affect that must be interiorised in order to be ethically effective. Moreover, the stigmatised nature of ‘venereal disease/s’ rendered practitioners' engagements with the topic problematic and added a further element of difficulty to the treatment of their patients.^ii^

Publishing medical theories and case notes was a widespread and effective means of self-promotion and soliciting business, although this function also necessitates careful scrutiny of them as evidence of doctor–patient interactions. It is highly probable that Turner embellishes his rate of patient retention and cure in the texts (and we unfortunately do not have any manuscript records from him), which would serve both to increase his professional reputation and to encourage compliance in readers who may themselves be future patients. Turner published *De Morbis Cutaneis* and *Syphilis* as he was using his past success as a surgeon to make the unusual move to a physician. He styles his prior practice as that of a ‘learned surgeon’, stressing his education in order to distance himself from the more stigmatised elements of that profession (ref. 11, pp. 211–12, ref. 12, p. 304). The skin also marked the theoretical boundary between the apprenticeship-trained surgeons' and university-educated physicians' external and internal jurisdictions, making *De Morbis Cutaneis* an apt publication. The line between ‘quack’ and legitimate medical practitioners was exceptionally blurry, thus Turner not only promoted his erudition, medical success and fashionable address but also attacked other healthcare providers as a means of shoring up his own position. His first major publication, *Apologia Chyrurgica* (1695), was an extended attack on quack practitioners, and he regularly stressed how his own ‘choice and try'd Remedies' were ‘approv'd by the constant Practice of good Authors, and confirm'd by our own’ (ref. 1, sig.S6^r^). He contrasts this against the rhetorical techniques of quacks and the hyperbolically diverse symptoms that they claim for venereal disease:

if your Head akes, it is the *Pox*; if you happen to be giddy, heavy or dull, faint or feeble in your Limbs, ‘tis all from the Pox: if the Palms of Hands are hot, ‘tis also from a Pocky Ferment. Nay, if your Urine does but stink of Piss, ‘tis still the *Pox*. (Ref. 2, sig.F6^r^. Original emphasis)

Turner opens *De Morbis Cutaneis* with a list of cited authors; since this does not include page numbers, it functions purely to emphasise his research credentials. As Steven Shapin has demonstrated, social capital fostered by such ‘gentlemanly practices’ was instrumental in establishing individual and collective credibility for the new science (ref. 13, p. xxi). Turner's readiness to shame quack practitioners is juxtaposed against his deferential consultations and disagreements with surgeons and physicians, where not shaming them (even if dead) is also a means of avoiding embarrassment for himself in being seen to go against a great name. In contradicting a point from Wilhelm Fabricius Hildanus (1560–1634), for example, he admits himself ‘very doubtful’ on a particular method, ‘and must herein dissent from that great and worthy Practitioner, to whose Memory notwithstanding I shall always pay the highest Deference and civil Veneration’ (ref. 1, sig.O1^r^). Illnesses themselves are pictured cowering before the wisdom of trained physicians, who can confront a chancre with a ‘Caustick [that] will humble its proud Looks and quickly level it’ (ref. 1, sig.P4^v^).

Turner directly styles his books as ‘Instructive to the young Practitioner’ (ref. 2, sig.K3^v^), and although medical texts were known to have a broader readership, would not necessarily have expected his anonymous patients to read their own case histories in his books. Where he indicts them in the texts as uncooperative or disrespectful, it is not to produce shame in the individuals, but to enhance his own professional image and sense of authority; as Michael Warner has noted, shame was understood as not requiring perception by the victim in order for the shamer to feel power (ref. 14, p. 290). Within the clinical encounters Turner records (to what extent we may believe his accounts) his use of shame reveals an adherence to the belief that, as his contemporary Ned Ward put it, the subject must be ‘Convicted by her own Conscience’ (ref. 15, sig.v.A3^v^). He advises young surgeons treating the pox that ‘what ever Way the Disease has been contracted’, it ‘behoves you’ to treat the patient's illness and leave ‘the Punishment of his Offence to the Checks upon his own Mind, or that Being against whose Mandates his Trespass was committed’ (ref. 2, sig.P5^r^). Privately, whether treating the pox or a less stigmatised disorder, Turner uses shaming rhetoric to increase his professional authority over patients and reads their compliance and cure as evidence of an honest contrition that can then be translated through print into a public performance. The widespread embrace of such rhetorical positioning and attacks on competitors, and the increasing power of Augustan physicians in the ‘medical marketplace’, produced a plethora of satirical representations attacking their vanity and shameful inattention to their patients' well-being (ref. 16, p. 148). Turner is therefore careful to present himself as not only in control but also exhaustively attentive to his patients' evolving symptoms and possessing a humility that renders him open to developing his own practice through research into preceding medical authorities and practical experience.

Healthcare was a highly diverse and competitive field with a limited code of medical ethics prior to ‘the professionalization of medical morality’ from the late eighteenth century (ref. 17, p. 853). Turner is particularly critical of individuals' ‘proud Boastings’ and ‘slye … Pretences’ against incurable diseases like cancer, where palliation would be more honourable (ref. 1, sig.G2^v^, ref. 18). Simultaneously, the physician must resist objections from ‘the common People’ that lead them to be overly ‘cautious’ in their interventions (ref. 1, sig.G1^r^). For Turner, this equates to a duty to promise no more or less than is medically possible to his patient. Turner includes some cases that did not achieve a perfect remedy, and in one instance insists that he is ‘not ashamed to let the Reader see, how I have been foyled’ in a pair of cases (ref. 1, sig.D3^v^). This objection is qualified, however, by his emphasised consultation with other practitioners, which renders the shame of the failure not merely his own—not only did he consult Sergeant-Surgeon Charles Bernard but another ‘Person pretending to a Secret for this Disease’ is also unable to offer an effective remedy (ref. 1, sig.D3^r^). Another elderly man comes to him with ‘a Pox of many Years standing’ that has left him with a ‘dripping rotten *Penis*’ (ref. 2, sig.Q1^r^. Original emphasis). Turner makes a point of calling in the surgeon William Petty, ‘being willing to have a Witness in what Condition this poor Fellow came out of the Quack's Hands’ before he attempts to treat him (ref. 2, sig.P8^v^). More damning is his self-reflection after convincing a female patient to let him excise a red birthmark from over her eyebrow, which proves more difficult than he had anticipated. He then remarks that, ‘Had I apprehended it so deeply rooted, I might not probably have been so very forward in the Undertaking, unless I had been solicited thereto by the Patient’ (ref. 1, sig.K3^v^). He attempts to exonerate himself by emphasising that he made sure to do his utmost once he had undertaken the treatment, rather than risking a relapse ‘to the Patient's farther Inconvenience and my own Discredit’ (ref. 1, sig.K3^v^). Joan Lane records that such gossip about the personalities, successes and failures of individual practitioners played a significant role in securing them further work and could circulate for several years after treatment (ref. 19, p. 220). Turner's concern for ‘discredit’ following an imperfect cure is developed further in his discussion of burns. In spite of the best efforts of even the most skilful practitioners, such injuries can leave the patient ‘unseemly and ill-favoured by the hard and rugged Scars’, which may even ‘rise up’ after a significant gap of time, ‘when the Work is done, and the Patient thinks he is come well off’ (ref. 1, sig.T2^v^). The surgeon's ‘bungling Patch-Work’ shows as disgracefully inferior to ‘the Creator's Workmanship’ as ‘the Puckering Work of a silly Child’ against ‘the finest Cambrick’ (ref. 1, sig.T2^v^). Turner offers an exhaustive and extremely defensive account of his treatment of a woman with severe burns to the face. He consults with another surgeon, warns the unmarried woman's parents ‘they must take her Face as happen'd’ (ref. 1, sig.T4^v^) and afterwards challenges ‘boasting Pretenders [to] undertake the like difficult Task, and leave smoother or handsomer Work behind them’ (ref. 1, sig.T6^r–v^). He then records with satisfaction the ‘Scorn and Indignation’ with which she dismisses a ‘young Novice’ and ‘villainous Oculist’ who offer to better his results (ref. 1, sig.T6^v^).

Within the doctor–patient interactions he records, Turner uses shame to attempt to compel patients towards what Talcott Parsons suggested as the two primary responsibilities of the person occupying the ‘sick role’: to try to regain health in order to return to their primary social role and to seek out and follow the instructions of ‘*technically competent* help’ (ref. 20, p. 437. Original emphasis). While preservation of God's creation and thus action for one's good health was a moral duty in early modern Britain, the choice of ‘competent’ help was more broadly defined—family and friends, local healers, wise women and empirics were widely consulted alongside surgeons and physicians (ref. 16, p. 152, ref. 21, p. 93). The eighteenth century saw a reframing of medicine as a body of knowledge restricted to a trained elite,^21–23^ and Turner's learned, Latinate style and overt denigration of amateur rivals accord with this shift. The usability of Parsons' model and the types of ‘sick role’ available in early modern Britain are subjects of ongoing investigation.^24–29^ As Olivia Weisser and Hannah Newton have demonstrated, people who became ill were allowed to retreat to the sickbed, to stop daily work, and so on, thus achieving the first of Parson's postulates (ref. 24, p. 105, ref. 25, pp. 173–75). Turner mentions an apothecary's apprentice whose master allows him to go home to his father's to be treated for a clap, and given two days a week for purging treatments after returning to employment (ref. 1, sigs.P5^r-v^). Where illness suggested a dishonourable dependence, however, fear and shame could compel sufferers who were not yet physically incapacitated to hide their symptoms, rather than withdraw and lose their place or become reliant on charity (ref. 25, p. 168, ref. 26, pp. 82–85). Alun Withey also highlights the gendered aspect of this requirement since for women the home was often also the workplace, rendering their withdrawal ‘less socially defined’ (ref. 27, p. 124). This would also include the many poorer people whose close living arrangements precluded separation from the rest of the household. Roy Porter and Dorothy Porter also note the social upheavals intrinsic to illness in the period that resulted in a significant stigmatisation of malingering (ref. 28, pp. 188, 192).

Turner's shaming of patients as a means of ensuring compliance is not restricted to those who are suffering from self-inflicted or otherwise stigmatised diseases such as the pox. Nor is it restricted to the patient in isolation—uncooperative parents of sick children are also fair game. Turner adhered to the belief that a mother's ‘imagination’ could affect her fetus (ref. 1, sig.H7^v^, ref. 30), thereby upholding a model of blaming mothers for congenital abnormalities (ref. 31, pp. 144–145). Children unable to offer useful information about their symptoms were notoriously difficult to diagnose and could be resistant to treatment (ref. 26, pp. 64, 71–2); the cooperation of parents and nurses was thus of great importance. Turner records a neighbour bringing his son to him with scabs on his scalp (ref. 1, sig.L8^v^). Though Turner diagnoses tinea, the father insists that ‘it was only a little Hurt from the Teeth of his Comb … and he wanted only a Bit of Plaister to heal it’ (ref. 1, sig.L8^v^). Turner is insulted by this request, as well as the father's disregard for his professional opinion, stating (presumably in front of the son) that if the father is ‘so very cunning [ie. knowledgeable]’ (ref. 1, sigs.L8^v^–M1^r^) he can treat the boy himself. Soon, however, the father must humbly return since, as Turner sneers, ‘notwithstanding all the good Wives Remedies’ that have been employed, the boy's condition has worsened (ref. 1, sig.M1^r^). Shame in front of superiors was acknowledged to be more powerful than the equivalent before equals or social inferiors (ref. 32, p. 35). By placing himself in a superior position to the father, as with his other patients, Turner therefore attempts to exacerbate the power inequity and use performative shame to greater effect.

For many, the stigmatised nature of the pox made them unwilling or unable to take on what benefits a ‘sick role’ may have afforded. Widespread confusion about the history, transmission and pathology of the disease was a key feature of its cultural identity and served to exacerbate the associated shame and fear. It was generally understood to have appeared in Europe at the close of the fifteenth century, where theologians were quick to describe it as a mass punishment from God. As early as the 1520s, however, the focus had shifted to its role as a personal punishment, especially for sexual sins (ref. 33, p. 261). A *lex talionis* sentiment held it apt that ‘the parts that Sin'd the most, most Torment felt’ (ref. 34, sig.D2^r^), and Turner echoes this in condemning a female patient's genitals as ‘fittest to suffer on Account of the wanton Use she had made of them’ (ref. 2, sig.R5^v^). Turner allowed for transmission in utero, through breast feeding, bed sharing or other close contact, but followed the majority opinion in concluding it ‘a venemous or contagious Distemper, for the most part contracted by impure Coition, or at least some Contact of the Genitals of both Sexes, or some other lewd and filthy Dalliance between each other that way tending’ (ref. 2, sig.B5^v^). The standard treatment in this period was salivation provoked by the topical application and/or ingestion of mercury, the side effects of which became as well-known and stigmatised as the bodily signs of the disease itself. This fed an immense market in quack medicines that stressed their discretion and efficiency. Related advertisements for barrenness and impotence treatments available at particular locations show how secrecy could depend upon sufficient affluence to employ a discrete servant (ref. 35, p. 49). Other vendors therefore promoted self-diagnosis and entirely postal transactions (ref. 36, pp. 209–210). Turner remained a firm adherent of mercurial treatments and was attacked for this increasingly unfashionable opinion later in his career (ref. 3, p. 170).

Turner acknowledges with frustration that many individuals are unable or unwilling to isolate themselves for long periods of mercury treatment. He praises a patient for having ‘strictly conform'd to the Rules prescrib'd, keeping his Chamber the whole time, which very much contributes to the Success of these Cures’, but acknowledges that ‘tis seldom that these People can have such Opportunity’ (ref. 2, sig.L2^r^). Turner therefore offers reassurance that, although confinement is preferable, ‘some (whose Business will not permit, unless perhaps an Hour after the Fume is over) have gone about their Affairs as usual’ (ref. 2, sig.K7^r^). In spite of Turner's threats, several customers in *Syphilis* leave his care prematurely, and he shames them as responsible for the return of their symptoms. Turner regularly confers with patients but never records their own speech, instead informing the reader of exchanges in retrospect, such as the case of a repentant recalcitrant who, he alleges, ‘was very sorry he had not been govern'd by my former Advice; for he found my Predictions were come to pass, and he could only blame his own Obstinacy and Indiscretion’ (ref. 2, sig.N8^v^). In comparison, good patients, ‘much fearing the lurking Snake’ continue with their treatments in spite of improved conditions (ref. 1, sig.C7^v^).

All medicine was moral in this period, and individuals were held responsible for maintaining the balance in their constitutions. Those who could not avoid disclosing illness or forsaking social responsibilities might attempt to exonerate themselves from culpability by highlighting their good moral character and social contributions, and attributing their illness to external factors (ref. 25, p. 168). Disease as derived from God—whether punitive or as a reminder of the need for humility—could also provoke shame from self and others (ref. 37, p. 46). Pox, attributed to sexual incontinence, was an exacerbated case; Turner thus offers contagion narratives for each of his patients with pox, especially women, for whom ‘honesty’ was by definition sexual.^38^ He shames several husbands who infect their wives by concealing their condition and/or resisting treatment. One patient hides the infection from his wife for four months, going ‘privately from one Quack to another’ until she works it out and sends for Turner (ref. 2, sig.M1^v^). Turner also reflects that ‘Women of the Town’ are at an advantage over ‘modest’ women infected ‘by an unkind or brutish Husband’: the former, operating under a different hermeneutics of suspicion about the body, recognise variations as signs of pox, ‘take the Alarm presently, seek out for Help, and … so soon as possible get rid of it’, whereas modest women mistake their symptoms for natural fluctuations in the body, or other lower-level disorders, and have ‘run into the last Degree of a *Pox*, before they knew what their Illness was, or look'd out for a proper Remedy’ (ref. 2, sig.C6^r^. Original emphasis). Honour could play a further role in diagnosis as physicians read the same symptoms as venereal or not depending on the patient's relationship to stigmatised risk groups: the author of *Medicina Flagellata* (1721) mocked doctors who read the same symptoms as scurvy in ‘sober discreet’ individuals, and as the pox in those ‘appearing inclined to Wantonness’ (ref. 39, sig.D1^v^). Turner reports patients whose symptoms appear venereal, but whom he is satisfied are ‘honest’, such as a man who is diagnosed with a clap by ‘one who call'd himself a Barber-Surgeon’: ‘the poor old Man, in great Anger, goes Home and tells his Wife how he has been abus'd, and she well satisfied of her own and her Husband's Honesty, came along with him to me … I pity'd the old Man’ (ref. 2, sig.Q1^v^). Conversely, he suggests that a woman who attempts to attribute her pox to wearing the clothing of an infected man to a masquerade, ‘perhaps was also after [the fashion of] the Masque, otherwise than by simply putting on the Habit’, suggesting that she has engaged in the sexual dissolution commonly attributed to these assemblies (ref. 2, sig.B6^r^).

Contending with the modesty and reticence of patients was a constant concern of practice, particularly since so much of diagnosis depended on their disclosure of symptoms and possible causes (ref. 16, p. 89). Even when a malady did not carry the pejorative connotations of a venereal disease, patients could be embarrassed by their bodies, perhaps according with Norbert Elias' proposal that the ‘civilising process’ necessitated increased concealment of the body's grosser parts and functions.^4^ Turner offers treatments to people suffering from excessively sweaty and smelly hands, feet, groin and armpits, lice, pimples, warts, dandruff, and so on. One young man comes to him with the pox, accompanied by his mother, and although evidently unconcerned that she knows this diagnosis, he feels compelled to hide himself physically: Turner records that ‘(she retiring for a Moment) I examin'd the *Penis*' (ref. 2, sig.P4^r^. Original emphasis). Female patients faced the added disadvantage of modesty comprising a key part of virtuous womanhood, with chastity determined by the maintenance of boundaries around the body (ref. 40, p. 52). Michael Stolberg suggests that physical examination was in fact not as rare a feature of early modern medical consultations as previously thought;^41^ however, Turner's *regular* examination of patients' bodies does seem unusual. This emphasis may reflect Turner's surgical background as surgeons were more known for emphasising their hands-on approach and treating the body from without (ref. 16, pp. 143, 174). For venereal patients, however, it may also reflect a bias against them as ‘discredited’ individuals whose testimonies are less trustworthy than other patients.^42^ Kevin Siena suggests that hospital records bear out Turner's belief that patients would sometimes seek treatment for the pox by pretending to possess a less stigmatising disorder (ref. 36, p. 205). Turner explicitly directs medical readers suspicious of a venereal disease in their patient to go ahead and treat it as such, regardless of the patient's denial: he is ‘no farther to strain this Point, but to proceed for [the patient's] Advantage, by the same Method, as if he had ingenuously acquainted us with the true Cause’ (ref. 2, sig.G3^r^). He also criticises doctors who ‘are very shy of these Enquiries’ and will not examine or press their patients for fear of insulting and ‘loosing [sic]’ them, especially when they are of a higher social standing than the physician (ref. 2, sig.G2^v^).

The hesitation Turner meets in some of his requests for examination, and the varying levels of negotiation he enters into with different patients, are indicative of his management and concessions to shame and power in his interactions with different classes of patients. For example, he treats a ‘very precise and exact Gentleman’ who on first consultation attributes pain and swelling in his genitals to ‘lying cross-legg'd in his Sleep’, denying all possibility of venereal infection (ref. 2, sig.L8^r^). At first, Turner allows the gentleman to determine his access to physical examination (‘with some Difficulty I was admitted to see’), but as the patient fails to improve, Turner develops a ‘Suspicion of the real Cause’, and he asserts his physical authority over the man's sickbed and body: ‘I observ'd his Linnen stained with a Running, which I show'd him; at the same Time taking hold of the *Penis*, that he had before used very industriously to conceal’ (ref. 2, sigs.L8^r–v.^ Original emphasis). Turner informs the now ‘weeping’ gentleman that this evidence confirms his professional opinion on the ‘Nature of his Disease’ and renders cure possible (ref. 2, sig.L8^v^). Turner's forceful physicality here is readily contrasted against the non-venereal case of a ‘couragious young Gentlewoman’ with a scabby growth on her forehead (ref. 1, sig.D2^r^). When he visits her, she tells him that she has similar marks over her body, and he asks to see them: ‘I desir'd she would show me her Elbows, and (if she pleas'd) her Knees … With the First she readily comply'd … [then] *she told me* the Tops of her Knees were rather worse … and the rest of her Body was perfectly free’ (ref. 1, sig.C8^r^, my emphasis). His concession that she should only show him her knees ‘if she pleas'd’ acknowledges, and may in fact have emphasised the possible immodesty in the act and the woman's embarrassment. After several visits, and the treatment having commenced under Turner and ‘an honest careful [female] Nurse’, Turner is able to view the woman's leg: ‘In turning down her Stockings by the Nurse, I prevail'd with the young Gentlewoman to let me take a View of one of her Knees, which she show'd me’ (ref. 1, sigs.C8^v^–D1^r^). That he waits until the nurse has uncovered her leg, rather than asking her to undress specifically, maintains propriety of both patient and physician in this encounter.

Turner also speaks of the need to treat patients in a manner mindful of their future exposure to bodily shame. Like many surgeons, he expresses concern about leaving visible scars, especially in the face, ‘where the utmost Diligence is wanted to prevent Deformity’ (ref. 1, sig.T1^r^). More unusually, he includes aesthetic concerns in his discussion of penile operations. Incisions in the foreskin are accompanied by an ‘inconvenience’ in that some patients ‘are after incommoded by the flagging Lips hanging down like the Thrills under a Cocks Throat’ (ref. 1, sig.P4^v^). This may not only cause embarrassment through physical difficulties in urinating or ‘the Use of Women’ but ‘at best being an Eye-sore, puts the Patient sometime after upon complyance with a Circumcision, by which he may be freed from farther Trouble’ although he must then contend with the derogatory (particularly anti-Semitic) associations of circumcision in this period (ref. 1, sig.P4^v^, ref. 43). One man, about to enter into his second marriage, seeks out Turner's surgical assistance for a minor aesthetic deformity of the penis that does not affect urinary or sexual function, and mid-operation, ‘in the Midst of his Pain he smil'd, saying it now look'd all of a Piece’ (ref. 1, sig.P8^r^). Turner, assisted by Bernard, operates on a 14-year-old boy with a severely obstructed urethra, cutting into his penis while servants hold him down and his mother watches (ref. 1, sigs.P6^v^–7^r^). Turner whispers to Bernard that they should also remove the ‘pendulous Lips’ of the foreskin, at which ‘the Boy over-heard us Talking, and cry'd out in Passion, he would die before we should cut again’ (ref. 1, sig.P7^r^). The boy's mother supports his decision, but Turner is concerned not only for the inconvenience this may cause him but also that it might subsequently ‘redound to my Discredit’ (ref. 1, sig.P7^r^). He ‘unwillingly’ complies with their desire to leave off cutting (ref. 1, sig.P7^r^) and does what he can with bandaging and liniments. The boy offers assurance that he can urinate satisfactorily, but Turner takes a startling glee in recounting that ‘I hear since that is apt to scatter, and (unless he takes Care and has Opportunity) to wet his Cloaths’ (ref. 1, sig.P7^r^). As with Turner's other ‘foolish’ former patients, shaming this child in print serves to promote his medical expertise.

Turner treated patients of all social classes and demonstrates an awareness of possible embarrassment for those too poor to access adequate medical care. In *De Morbis Cutaneis*, he offers a remedy for haemorrhoids composed of sitting in warm milk and oil of poppies or violets, and adds that ‘for poor People Flannels wrung out of the same, prepar'd in lesser Quantity may suffice’, thus enabling them to access treatment for a fraction of the apothecary's fee (ref. 1, sig.Q3^v^). In *Syphilis,* he changes from daily visits to twice-weekly for a long-term patient who can no longer afford the expense and treats another ‘Poor, but honest Woman’ poxed by her husband for free, ‘purely as an Act of Charity’ (ref. 2, sig.N2^r–v^). Elsewhere a merchant patient resists salivation, ‘having much Business upon his Hands’ (ref. 2, sigs.M2^r–v^). In contrast to the elite men whose ‘performative libertinism’ might incorporate a consciously shameless approach to diseases like the pox^44^—such as appears in the coterie poetry of John Wilmot, Earl of Rochester—this patient highlights the economic importance of sexual honour for the middling sort in declaring that he ‘would by no means risque his Reputation (on which a handsome Living was depending)’ (ref. 2, sigs.M2^r–v^). Turner does not treat any elite figures in *Syphilis*, but his comments on such ‘libertines’ are mild; for example, he announces the condom to be ‘the best, if not only Preservative our Libertines have found out at present; and yet, by reason of its blunting the Sensation, I have heard some of them acknowledge, that they had often chose to risque a *Clap*, rather than engage *cum Hastis sic clypeatis* [with spears thus sheathed]’ (ref. 2, sig.F5^r^. Original emphasis). His emphasis is predominantly on his own wit, rather than castigation.

Nevertheless, Turner's representation of the libertines as choosing to ‘risque’ their health is echoed across critiques of shamed behaviours. His emphasis on the ‘innocent’ contagion of deluded wives, and so on, also acknowledges, and in some senses exacerbates, the stigmatisation of less innocent contractors (ref. 45, p. 42). Turner employs some of the same terminology as moral campaigners, alluding to treatment that includes restrictions of diet, movement and sexual activity as an unpleasant ‘Penance few of our People will submit to’, and that by the ‘good Discipline’ and constriction of a salivation ‘their Appetites are soon taken off from their beloved Vices, and all those other Debauches’ (ref. 2, sig.H5^v^). In diagnosis and treatment, Turner privileges the use of forethought and the expense of time initially where it will save it in the long term. This is applicable to both the surgeon and patient, who share the responsibility for ‘verify[ing] not an old Proverb, *More Haste, worse Speed*’ (ref. 2, sig.H3^r^. Original emphasis). The fast treatment might not only prove medically inadequate but also fail to allow sufficient time for the patient to consider the actions that brought him or her to this impasse. Turner admonishes those who do not offer a performative contrition, reproaching a former patient for ‘rather glorying in his Shame, than endeavouring to conceal his Folly’ by showing pieces of his syphilitic bone in a box as a boast to friends (ref. 2, sig.O7^r^). The phrase ‘glorying in shame’ originates in Philippians 3:19 and recurs across early-modern texts as a severe condemnation of the abrogation of dominant morality.^iii^ For some patients, such prompts to shame as Turner provided may have satisfied desire for a penitential component of their treatment as important as any medication.

For all practitioners discussing venereal diseases, it was of course in their best interests to limit the patients' stigmatisation, and thus discount possible ‘courtesy stigma’ attaching to themselves.^42^ Turner adds that, although it is useful to overcome patients' shame sufficiently to gain their full disclosure of symptoms, establishing too great a trust leading to familiarity risked passing on stigma to the physician. In closing the preface to the extended 1724 edition of *Syphilis*, Turner warned: ‘although I would have you be their *Confidents*, I must dissuade [young practitioners] by all Means from being their *Companions* … [or] you will hereby make your selves mean, be despised of all those of Reputation’ (ref. 46, sig.A8^r^. Original emphasis). Turner does not challenge the stigmatisation of pox, but nevertheless castigates patients who attempt to hide their disease and its cause, especially when it puts his own reputation at risk. He berates one patient for ‘his Modesty, or rather Folly, in concealing his Case’, and causes him to beg for discretion: ‘He coloured and presently fell a weeping, conceiving well it would avail him nothing to deny it, but begg'd of me to be careful of his Reputation, which was considerable among his Party, and which I promis'd him that I would’ (ref. 2, sigs.L8^v^–M1^r^). Doctor–patient confidentiality was not yet an axiomatic feature of practice, rendering Turner's promise of secrecy necessary (ref. 36, p. 211). His ability to provide follow-up care or an account of the success (or not) of individual cases is hampered by patients who ‘advancing the Reward beforehand, kept themselves *Incognito*’ (ref. 2, sig.F2^r^), including one woman who insists on remaining masked (ref. 2, sig.O7^v^). Such cases also help us to understand why ashamed patients may have remained with Turner despite his castigations, rather than increase their risk of exposure by consulting multiple practitioners.

In another venereal case, Turner comprehensively undermines the woman's attempts to maintain modesty as a means of forcing her complete submission to his regime. After scarifying and dressing her severely ulcerated genitals, Turner proposes returning each day to change the dressings. The woman declines, suggesting that she will be more comfortable if her mother performs the task: ‘this modest Creature seeming asham'd to be thus expos'd every Day, now the Danger was past, desir'd I would leave Dressings for her Mother to put on’ (ref. 1, sig.R6^r^). Recounting this case at its conclusion, Turner's use of ‘modest’ is revealed to be intended ironically. Despite Turner leaving detailed instructions, he says, ‘being perfectly easie, [she] arose daily and went abroad, by which her Dressings felling into Wrinkles, were apt to slip off, and in one Night's Time … by her Negligence, the Parts were growing fast together’ (ref. 1, sig.R6^r^). She and her mother are proven to be careless and negligent, and Turner compels her to beg for treatment and to promise chastity:

I ask'd jestingly, if she was content, provided we took Care to secure a Passage for her Urine, to let the other Part remain as it was; which (to hear what she would say) I told her could not now be parted without a great deal of Pain: She beg'd I would assist her, and divide with as little Pain to her as possible, promising to be more careful as well as chast for the Future. (Ref. 1, sig.R6^r^)

Turner's assistance is predicated on the woman's performance of shame and contrition, enacted in submission to his medical authority and renunciation of previous sexual immodesty.

In *De Morbis Cutaneis* and *Syphilis*, Turner demonstrates the precarious relationship to shame occupied by everyone involved in early eighteenth-century healthcare. Printed at a transitional point in his career, *De Morbis Cutaneis* in particular, with its ostentatious display of learned reading, represented Turner's conscious project to prove himself worthy of the membership of the College of Physicians, and physicians deserving of their elevated position in the medical marketplace. His performative humility before the sick body and received medical authority needed to be balanced with the denigration of alternative healthcare perspectives from informal healers that abounded in the period. The physician crafted and managed his own reputation as a moral and medical authority who could treat all distempers. Though this had to include venereal diseases, it could not be seen as condoning or facilitating the shameful behaviours associated with a sexual illness. Turner thus devotes significant portions of his texts to managing patients' reticence over disclosure of symptoms, expectations for cure and impudence towards medical authority. Turner advises trainee surgeons and physicians to treat patients with pox regardless of their stigmatisation, with the understanding that they will instead be subject to self-imposed castigations that can provoke performative shame. The patients' adherence to the doctor's strict regimens can serve as evidence of this contrition and their desire to return to the morally elevated position of ‘good health’. Conversely, resistance to the physician's directions testifies to the patient's lack of interiorised shame and ethical development.

## Footnotes

i
*De Morbis Cutaneis* was revised and reprinted in 1723, 1726, 1731 and 1736. It was translated into French (1743) and German (1766) (ref. 3, p. 72). *Syphilis* was expanded and reprinted in 1724, 1727, 1732 and 1737. I have chosen to use the first editions of these texts for a better comparison at this transitional stage of Turner's career.

ii The ‘venereal disease’ in Turner's text is the pox, which he considers to be the ‘confirmed’ or second stage of a ‘clap’ (gonorrhoea). He does not refer to the disease as ‘syphilis’ outside the book's title. I follow other historians of medicine in using early modern terms (particularly ‘pox’) to distinguish it from the specific modern disease and to highlight its social construction.^8–10^

iii Philippians 3:18–19 (King James Version): ‘(For many walk, of whom I have told you often, and now tell you even weeping, that they are the enemies of the cross of Christ: Whose end is destruction, whose God is their belly, and whose glory is in their shame, who mind earthly things.)’
